# DUF99 family proteins are novel endonucleases that cleave deoxyuridine on DNA substrates

**DOI:** 10.1016/j.jbc.2024.107901

**Published:** 2024-10-18

**Authors:** Jinquan Li, Runyue Xia, Wen-Cong Huang, Jiazheng Gu, Meng Li

**Affiliations:** Archaeal Biology Centre, Synthetic Biology Research Center, Shenzhen Key Laboratory of Marine Microbiome Engineering, Key Laboratory of Marine Microbiome Engineering of Guangdong Higher Education Institutes, Institute for Advanced Study, Shenzhen University, Shenzhen, China

**Keywords:** endonuclease, DUF99 protein, *Haloferax volcanii*, domain of unknown function, DNA deamination

## Abstract

DNA deamination occurs constantly in a cell and causes DNA damage. As this damage can be deleterious, organisms have evolved many systems to eliminate it, such as Endonuclease V (Endo V). DUF99 family protein contains a domain of unknown function similar to Endo V but has not been experimentally characterized to date. Here, we show that DUF99 family proteins cleave the 3′-side of deoxyuridine (dU) on DNA substrates. Based on phylogenetic analysis, we designated this new protein family as Endonuclease dU (Endo_dU). We also observed that Endo_dU coding gene frequently colocalizes with that of uracil-DNA glycosylase (UDG) in halophilic archaea, and we further performed gene knockout of *Endo_dU* gene on *Haloferax volcanii.* The transcription level of *UDG* gene on *Endo_dU* knockout strain was increased when induced by sodium bisulfite. Thus, we hypothesize that Endo_dU establishes a new endonuclease family with broad phylogenetic distribution and may participate in DNA repair.

DNA damage occurs constantly in a cell due to intrinsic and extrinsic environmental factors, which lead to mutation, cancer, and even cell death (1). DNA deamination is a typical example of damage. Deamination of cytosine, guanine, adenine, and 5-methylcytosine results in the formation of uracil, xanthine, hypoxanthine, and thymine, respectively. When present in DNA, uracil tends to pair with adenine. Hence, a cytosine to uracil change results in a conversion of a G:C base pair to an A:T base pair during DNA replication (2). Consequently, organisms have evolved several DNA repair pathways that eliminate genetic lesions to maintain genome integrity.

The main DNA repair pathways include base excision repair (BER), nucleotide excision repair (NER), alternative excision repair (AER), and mismatch repair (MMR) for single-strand damage (3, 4, 5, 6, 7). Among them, BER and AER are the major pathways that repair deaminated bases. The BER pathway is initiated when DNA glycosylases recognize and then remove the damaged base, with the formation of an apurinic/apyrimidinic (AP) site (8). Uracil-DNA glycosylase (UDG) removes misincorporated uracil from DNA by cleaving the N-glycosidic bond of the damaged base (9). However, UDG lacks an AP lyase activity to convert a base lesion into a single-strand break. By contrast, AER is initiated directly by a single nick near the site of DNA damage, followed by downstream processes to repair the lesion (6). Among endonucleases involved in AER, endonuclease V (EndoV) homologs are highly conserved in the three domains of life (Archaea, Bacteria, and Eukarya). These enzymes primarily recognize and cleave the second phosphodiester bond on the 3′-side of the deoxyinosine or inosine (hypoxanthine attached to a deoxyribose or ribose sugar ring) on DNA/RNA, and are involved in nucleic acid metabolism (10, 11, 12, 13, 14).

The development of next-generation sequencing technologies and the explosion of metagenomic data allowed the identification of many hypothetical proteins that lack annotations. These proteins share little sequence homology with previously annotated proteins. They contain domains of unknown function (DUF) whose biological function awaits experimental characterization (15). DUF proteins represent 4244 families registered in the Pfam database (http://pfam.xfam.org, version 33.1), accounting for over 23% of all Pfam-registered families (18,259 families, as of June 2020) (16). Among DUF protein families, 16% (679 families) are only found in prokaryotes, 30% (1273 families) are only found in eukaryotes, and 8% (340 families) are found in all domains of life. The biological roles of these proteins are primarily informed by sequence homology analysis and structure determination (17, 18). Although these methods shed some light on protein functions, experimental characterization of enzymatic activity is needed for full protein evaluation (19).

DUF99 family (Pfam family PF01949) contains 2185 proteins, as registered in the Pfam database (16). Members of this family are found in archaea and bacteria, and also in some eukaryotes. Considering their wide taxonomic distribution, the activities of DUF99-containing proteins are most likely biologically essential but await experimental verification (20). Based on previous annotations, DUF99 proteins share sequence homology with the EndoV superfamily, with a secondary structure with a typical ribonuclease (RNase) H-like fold (21). However, the biological functions of DUF99 proteins have not yet been determined, although a crystal structure of DUF99 protein from *Archaeoglobus fulgidus* was solved in 2007 (http://www.rcsb.org/structure/2QH9).

In this study, we evaluated the evolution and enzymatic activity of DUF99 domain-containing proteins. The phylogenetic analysis showed that these proteins form a monophyletic group distinct from other endonucleases. Further, we discovered that they target both single-stranded (ss) and double-stranded (ds) DNA substrates, containing deoxyuridine (dU), specifically cleaving the 3′-side of dU. We thus designated this novel endonuclease family as endonuclease dU (Endo_dU). Strikingly, we found some prokaryotes encode the genes for Endo_dU and known endonucleases, such as UDG, in the same operon. The knockout of *Endo_dU* gene on *Haloferax volcanii* (*H. volcanii*) increased the transcription level of *UDG* gene in the presence of base deamination inducer, sodium bisulfite (SB). Taken together, our study reveals the enzymatic activity of DUF99 domain-containing proteins as well as establishes a novel endonuclease family. We propose that Endo_dU homologs may be involved in DNA metabolism in some microorganisms, and could constitute a new tool for nucleic acid manipulation and relevant applications.

## Results

### Phylogenetic analysis of DUF99 proteins

**Figure 1:**
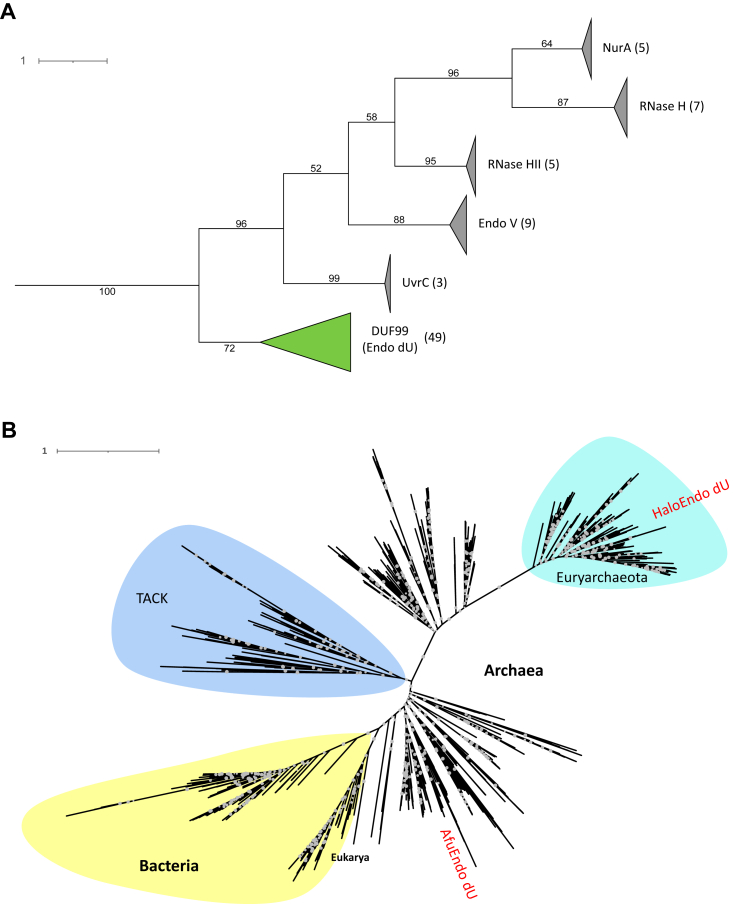
**Phylogenetic analysis of DUF99 proteins.***A*, maximum-likelihood phylogenetic tree of DUF99 proteins with RNase H-like domains of EndoV, UvrC, NurA, RNase H, and RNase HII. The phylogenetic tree was inferred using IQ-TREE with LG + G model. Bootstrap values are shown at each node. The numbers in brackets refer to the number of sequences in the collapsed clades. See Fig. S7 for details. *B*, unrooted maximum-likelihood phylogenetic tree of DUF99 proteins. The phylogenetic tree was inferred using IQ-TREE. Gray dots indicate bootstrap values larger than 70. See Fig. S8 for details.

A BLAST search of the NCBI nr protein sequence database using annotated *A. fulgidus* DUF99 protein as a query identified 2359 DUF99 homologs (E-value < 0.05). Most of these were sequences of archaeal and bacterial origin. In contrast to the wide distribution of DUF99 homologs in prokaryotes, the search identified only two homologs in eukaryotes, such as *Rhodophyta*. We then performed a phylogenetic analysis of DUF99 proteins with several representative RNase H-like superfamily members (21), such as EndoV, UvrC, and RNase HII. In the maximum-likelihood phylogenetic tree, the DUF99 protein family formed a monophyletic group that did not cluster with other RNase H-like proteins and was distinct from EndoV proteins (Fig. 1*A* and Fig. S1). This indicated that DUF99 proteins have arisen as a novel endonuclease family possibly involved in DNA metabolism.

We also constructed an unrooted maximum-likelihood phylogenetic tree for the DUF99 protein family to illustrate its distribution and evolution. The topology of the phylogenetic tree generally did not follow that of the 16S rRNA gene tree. Bacterial DUF99 proteins formed a monophyletic group with a bootstrap value of 92.5. Bacterial and “Thaumarchaeota-Aigarchaeota-Crenarchaeota-Korarchaeota (TACK)” superphylum subset of archaeal homologs were separated by a long branch, with a bootstrap value of 100, from archaeal “Euryarchaeota” phylum homologs (Figs. 1*B* and S2). The two DUF99 proteins from *Rhodophyta* clustered closely with the bacterial group, and other archaeal homologs were scattered in the tree. Notably, the “Euryarchaeota” monophyletic group, mainly consisting of halophilic archaea, formed a well-supported clade with a bootstrap support value of 100. These results suggested that DUF99 proteins may contain different subtypes and have evolved independently in different species.

### Cleavage properties of DUF99 proteins with ssDNA substrate

**Figure 2:**
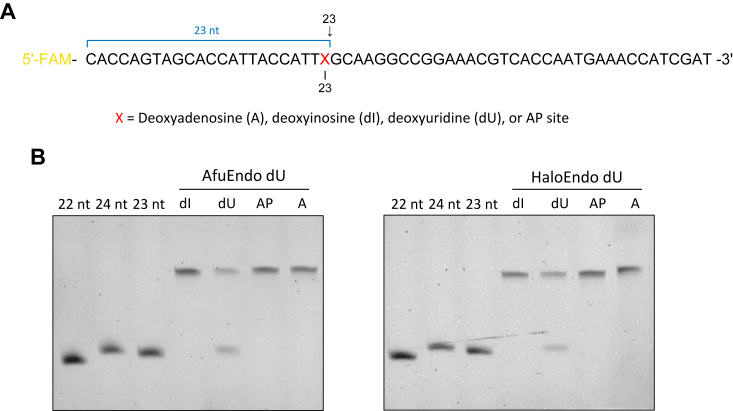
**Cleavage activities of Endo_dU toward different ssDNA substrates.***A*, schematic of the ssDNA substrates used in this experiment. The cleavage site is indicated by the arrow above, resulting the cleavage product 23 nt in length. The numbers below indicate the position of nucleotide counting from the 5′-end. *B*, AfuEndo_dU and HaloEndo_dU cleaved different ssDNA substrates. 30 nM 5′-FAM-labeled ssDNA substrates were incubated with 300 nM AfuEndo_dU or HaloEndo_dU at 37 °C for 30 min. The products were resolved by 8 M urea–15% denaturing PGAE. dI, ssDNA substrate containing a deoxyinosine (dI) at position 23; dU, ssDNA substrate containing a deoxyuridine (dU) at position 23; AP, ssDNA substrate containing an AP site at position 23 A, undamaged ssDNA substrate with a deoxyadenosine at position 23.

We successfully expressed and purified DUF99-domain-containing proteins from *A. fulgidus* (AfuEndo_dU), *Halobacterium* sp. DL1 (HaloEndo_dU), Gammaproteobacteria bacterium (GmEndo_dU), *Candidatus* Lokiarchaeota archaeon (LokiEndo_dU), and *Candidatus* Thorarchaeota archaeon (ThorEndo_dU) in *Escherichia coli* (*E. coli*). Notably, we could not obtain AfuEndo_dU proteins from lysate which was incubated at 65 °C before being applied to a chromatography (data not shown). Since EndoV, a DUF99 homolog, primarily recognizes and cleaves deoxyinosine (dI) on ssDNA, we first tested DUF99 protein cleavage activity using fluorescently labeled ssDNA substrate containing dI at position 23 relative to the 5′-end (Fig. 2*A*). However, we did not detect any cleavage products in reactions with any of the DUF99 proteins tested. We next tested the endonuclease activity toward other substrates with modification, such as dU and an AP site. Intriguingly, DUF99 proteins cleaved an ssDNA substrate containing dU, resulting in the release of a 23-nucleotide (nt) fluorescently labeled cleavage product (Figs. 2*B* and S3).

**Figure 3:**
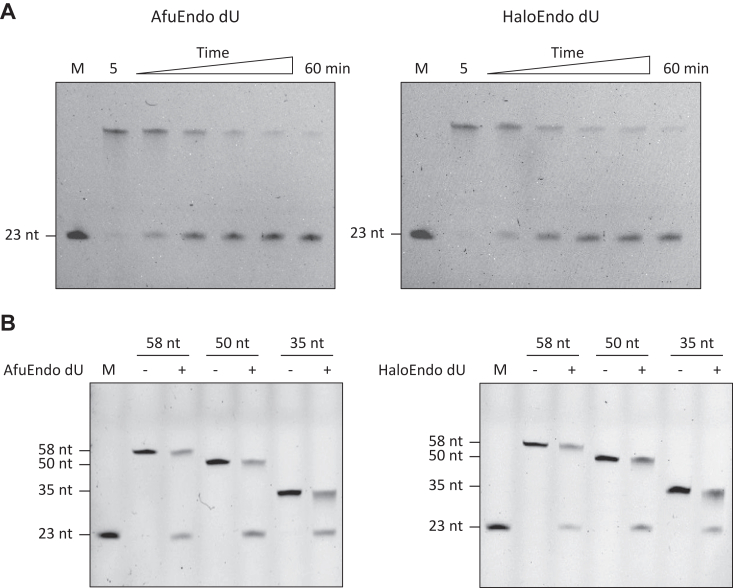
**Endonuclease activities of Endo_dU toward dU-containing ssDNA substrates.***A*, Endo_dU proteins (300 nM) cleaved the dU-containing ssDNA substrates in a time-dependent manner. The dU-containing ssDNA substrates (30 nM) were incubated with AfuEndo_dU or HaloEndo_dU at 37 °C. The reaction mixtures were removed at various time points (5, 15, 30, 40, 50, 60 min), quenched by mixing with gel loading buffer. *B*, Endo_dU proteins (300 nM) cleaved various lengths of ssDNA substrates (30 nM) at 37 °C for 30 min. The lengths of ssDNA substrates were indicated above, with a dU at position 23. M, 23 nt ssDNA marker.

Analysis of AfuEndo_dU and HaloEndo_dU revealed that these two proteins specifically cleave dU-containing ssDNA in a time-dependent manner (Fig. 3*A*), but not normal DNA or DNA containing other base modifications. Further, the enzymes did not cleave RNA substrates (Fig. S4). To confirm the cleavage properties of DUF99 proteins, we then tested their endonuclease activity using a set of synthetic dU-containing ssDNA of different lengths (Table S1). The analysis revealed that AfuEndo_dU and HaloEndo_dU cleave dU-containing ssDNA of various lengths, longer than 35 nt, generating 23-nt fluorescently labeled cleavage products (Fig. 3*B*). Taken together, considering the protein specificity toward dU-containing DNA, we designated this novel endonuclease family as the Endonuclease dU (Endo_dU) family.

### Optimal cleavage conditions for Endo_dU

**Figure 4:**
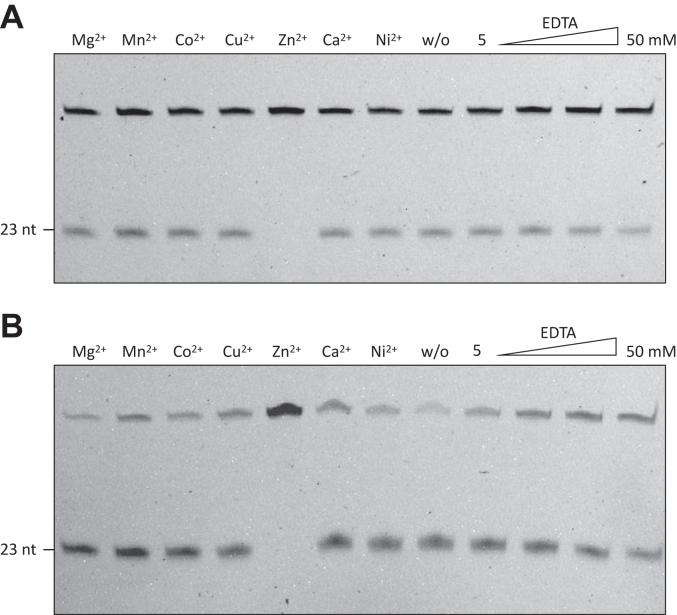
**Effects of divalent metal ions on the endonuclease activity of Endo_dU.***A*, 300 nM AfuEndo_dU and (*B*) HaloEndo_dU cleaved 30 nM dU-containing ssDNA substrates using various metal ions (5 mM) and increasing concentration (5, 10, 20, 50 mM) of EDTA.

We next moved to determine the optimal cleavage conditions for AfuEndo_dU and HaloEndo_dU, representing two distinct clades of Endo_dU in phylogenetic analysis (Figs. 1*B* and S1). Since most endonucleases require divalent metal ions to function, we first investigated the effects of metal ions (Mg^2+^, Mn^2+^, Co^2+^, Cu^2+^, Zn^2+^, Ca^2+^, and Ni^2+^) on their activity. Notably, while the enzymes were active in the presence of various metal ions, they were also active in the presence of high concentrations of the metal-ion chelator EDTA (Fig. 4, *A* and *B*). We noticed that protein was easily precipitated in the buffer with Zn^2+^, thus losing the cleavage activity. Therefore, they could be metal cation-independent endonucleases.

**Figure 5:**
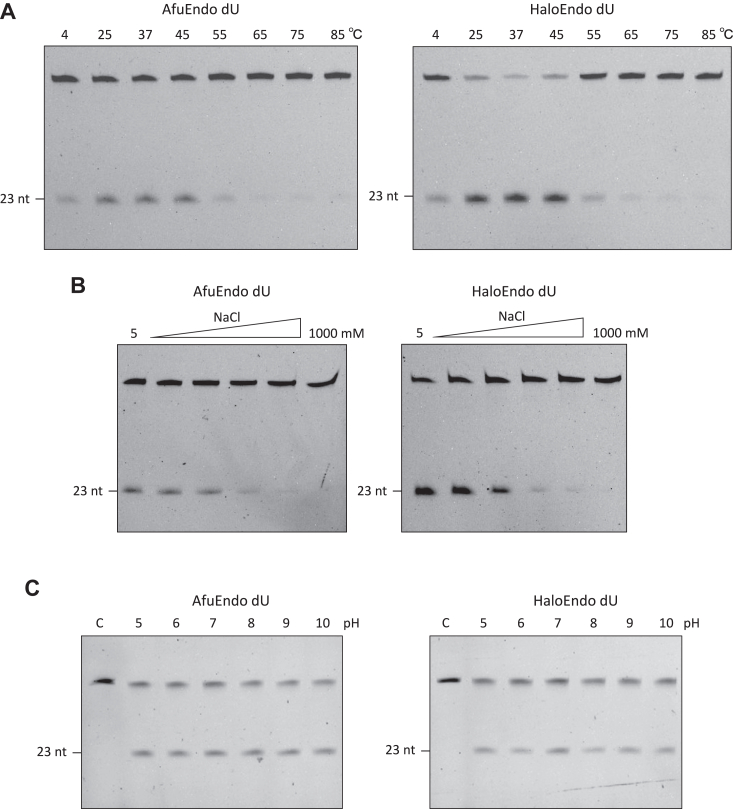
**Optimization of cleavage conditions of AfuEndo_dU and HaloEndo_dU.***A*, effects of temperature on the endonuclease activities. Cleavage reactions were performed at indicated temperatures for 30 min. *B*, effects of NaCl concentration on the endonuclease activities. The reactions were performed in reaction buffer with increasing concentrations (50, 100, 200, 400, 800, 1000 mM) of NaCl. *C*, effects of pH on the endonuclease activities. The reactions were performed in a reaction buffer with varied pH units indicated above but otherwise identical components. *C*, control group without adding the protein. The above reactions were performed using 300 nM proteins and 30 nM DNA substrates at 37 °C for 30 min.

Next, we determined the optimal temperature range at which AfuEndo_dU and HaloEndo_dU are active, by testing their cleavage efficiency in the 4 to 85 °C range. Both endonucleases were active in the 4 to 55 °C range, with the optimal reaction temperatures between 25 and 45 °C (Fig. 5*A*). Although AfuEndo_dU is derived from a thermophilic microorganism, which grows between 60 and 85 °C, the enzyme exhibited little cleavage activity at high temperatures. We also investigated the thermostability of AfuEndo_dU and HaloEndo_dU, by incubating the proteins for different amounts of time at different temperatures before assaying. Both proteins retained the endonuclease activity after less than 60 min incubation at 37 °C. However, pre-incubation at 65 °C for over 5 min completely abolished their activity (Fig. S5).

We then tested the effects of ionic strength and pH on the endonuclease activity of AfuEndo_dU and HaloEndo_dU. We tested the former by assaying in the presence of various concentrations of NaCl (50–1000 mM). The analysis revealed that the proteins exhibited cleavage activity in the presence of up to 200 mM NaCl, with the increasing NaCl concentrations inhibiting their activity (Fig. 5*B*). We tested the effects of pH by varying the pH of the reaction buffer. Both proteins were active over a wide pH range, from 4 to 10 pH units (Fig. 5*C*).

### Cleavage properties of Endo_dU with dsDNA substrate

**Figure 6:**
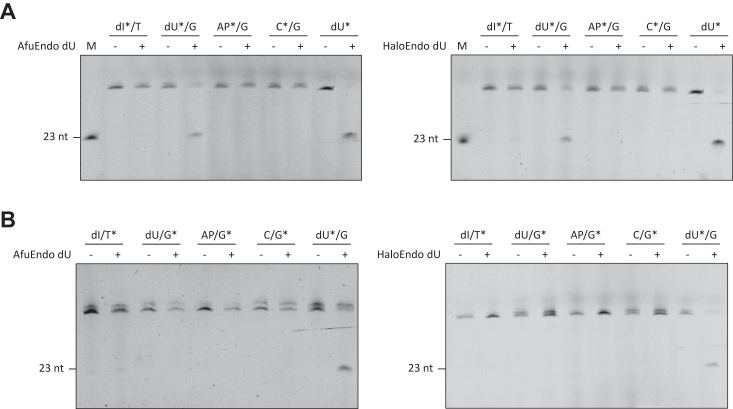
**Endonuclease activities of Endo_dUs toward dsDNA substrates.***A*, substrates with 5′-FAM labeled (30 nM) on the lesion strand were subjected to reactions with AfuEndo_dU and HaloEndo_dU (300 nM). *B*, substrates with 5′-FAM labeled on the opposite strand of the lesion were subjected to reactions with AfuEndo_dU and HaloEndo_dU. The labeled strands are indicated by asterisks. M, 23 nt ssDNA marker. The cleavage reactions were performed at 37 °C for 60 min.

We next tested the ability of AfuEndo_dU and HaloEndo_dU to cleave dsDNA substrate. The proteins cleaved a dU-containing DNA strand fluorescently labeled at 5′-end (Fig. 6*A*), but not the complementary strand fluorescently labeled at 5′-end, as determined in independent experiments (Fig. 6*B*). Notably, the multiple bands substrate showed in gels could be due to incomplete denaturation since dsDNA was easily annealed during electrophoresis.

We also examined whether the proteins cleave dsDNA containing a base-pair mismatch. We tested that by performing cleavage reactions with dsDNA containing different mismatched base pairs (A/A, A/C, A/G, C/C, C/T, G/G, G/T, and T/T) as substrates. We did not detect any cleavage activity with dsDNA containing mismatched base pairs (Fig. S6), which suggests that these proteins are not mismatch-specific endonucleases, but dU-specific endonucleases that only cleave dU-containing ssDNA or dsDNA.

### Sequence and structural analysis of Endo_dU

**Figure 7:**
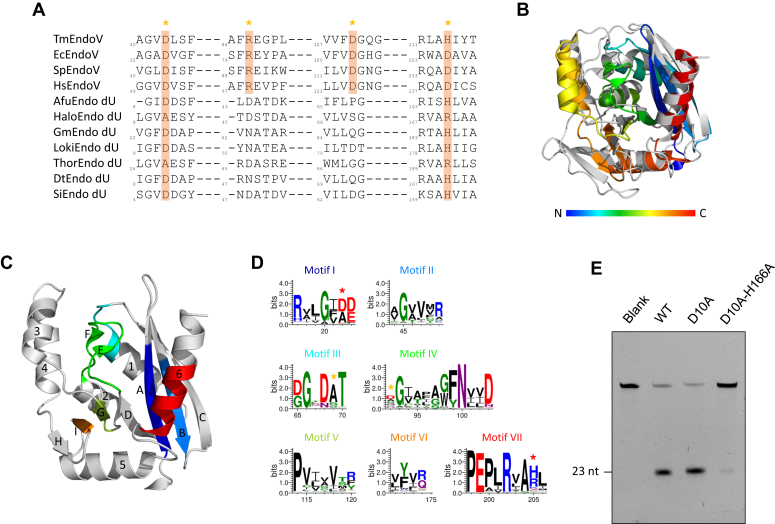
**Comparison of Endo_dU with EndoV and analysis of the highly conserved regions of Endo_dU.***A*, multiple sequence alignment of AfuEndo_dU, HaloEndo_dU, GmEndo_dU, LokiEndo_dU, ThorEndo_dU, Endo_dU of *Deinococcus-Thermus bacterium* (DtEndo_dU, NCBI accession no. RMH56893.1), and *Sulfolobus islandicus* (SiEndo_dU, NCBI accession no. WP_012953046.1) with TmEndoV, EndoV of *Escherichia coli* (EcEndoV, NCBI accession no. PSG80310.1), *Schizosaccharomyces pombe* (SpEndoV, NCBI accession no. CAA93810.1), and *Homo sapiens* (HsEndoV, NCBI accession no. NP_775898.2). Conserved DEDD residues of EndoV are highlighted and indicated by the asterisks above. See Supplementary Materials (Fig. S9) for the full result. *B*, superposition of the structure of AfEndo_dU (rainbow color, *blue* at the N-terminus to *red* at the C-terminus) and the structure of TmEndoV (*gray color*). *C*, cartoon representation of AfuEndo_dU (*gray color*). Seven highly conserved motifs are colored individually as shown in (*D*). α-helices and β-strands are numbered according to the order in sequence. *D*, WebLogo analysis of seven conserved motifs within Endo_dU. Sequence logos were generated using the multiple sequence alignment result from the phylogenetic analysis, and visualized by WebLogo tool (http://weblogo.threeplusone.com/). Residues at the corresponding positions of the conserved DEDD residues of EndoV are indicated by asterisks above. See Supplementary Materials (Fig. S10) for the sequence logos of the full-length Endo_dU. *E*, endonuclease activities of AfuEndo_dU and its mutants toward dU-containing ssDNA substrates. The cleavage reactions were performed at 37 °C for 30 min.

Endo_dU shares some functional and structural similarities with EndoV, a well-studied family of enzymes. Therefore, to investigate the structural basis of DNA cleavage by Endo_dU, we compared the amino acid sequences and structures of Endo_dU and EndoV family members. Multiple sequence alignments revealed that Endo_dU proteins harbor an incomplete conserved DEDD catalytic tetrad of EndoV at positions corresponding to those of EndoV (Fig. 7*A*). Further, the structure of AfuEndo_dU (PDB ID:2QH9) shows a typical RNase H-like fold, with a core structure of five β-strands and three α-helices (Figs. 7*C*, S7). Compared with the structure of a well-studied TmEndoV (PDB ID: 2W35), AfuEndo_dU lacks the first N-terminal α-helix. Further, in AfuEndo_dU, the sixth β-strand of TmEndoV is replaced by two α-helices, with a configuration similar to that of the first α-helix of EndoV (Fig. 7*B*).

Alignment of non-redundant sequences of Endo_dU proteins revealed several motifs, motifs I to VII, highly conserved within the Endo_dU family (Fig. 7, *C* and *D*). Motifs I and II form the structural backbone of the first two β-strands. The first conserved aspartic acid residue found in DEDD-type nucleases lies on βA but is not conserved in the Endo_dU family. Motif III consists of highly conserved glycine, aspartic acid, and threonine within L_C1_ (the loop between βC and α1) and α1. Motif IV lies on L_D2_ (the loop between βD and α2); short βE and βF are present in this loop region in some homologs (Fig. S7). Motifs V and VI consist mainly of several hydrophobic residues on βG and βI, forming a putative uracil-binding pocket together with motifs IV and VII. This putative uracil-binding pocket is highly conserved in these proteins, according to ConSurf server calculations (Fig. S8). Motif VII forms the structural backbone of the final α-helix, with a positively-charged residue (histidine, arginine, or lysine) at a position corresponding to that of the fourth conserved aspartic acid in DEDD-type nucleases.

We next constructed AfuEndo_dU variants with alanine substitutions of several residues predicted to be important for protein function (Fig. S11). The substitution of the first conserved negatively charged residue, D10A, did not affect the cleavage activity. While D10A-H166A double substitution dramatically decreased the endonuclease activity (Fig. 7*E*). These observations suggest that Endo_dU proteins do not utilize the conventional DEDD catalytic tetrad to coordinate metal ions for activity, whereas the last conserved H/D/R residues are important. However, their detailed cleavage mechanism remains to be explored.

### Endo_dU expression *in vivo*

**Figure 8:**
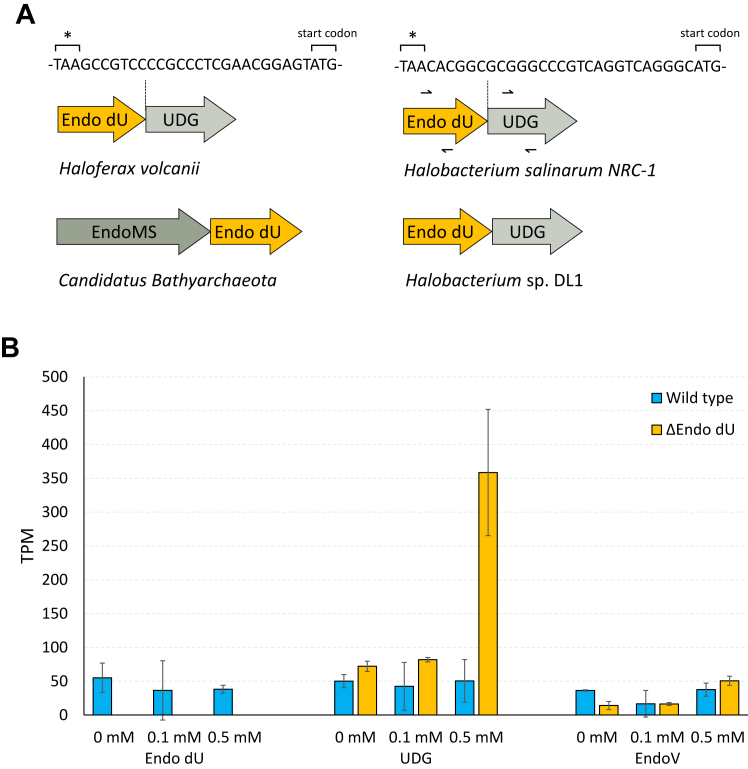
**Study of gene transcription levels *in vivo*.***A*, schematic of the locus containing Endo_dU and its neighbors. *B*, TPM of each gene induced by different concentrations of SB in wild type and *Endo_dU* knockout (Δ*Endo dU*) strain.

According to bioinformatic analysis, the genes encoding Endo_dU in many halobacteria often colocalize with those of UDG, with a similar genomic arrangement to that of genes of some endonucleases involved in DNA repair (22). For example, an *UDG* gene is located 24 bp downstream of the adjacent *Endo_dU* gene in the genome of *H. volcanii*, 27 bp downstream in the genome *H. salinarum* NRC-1 (Fig. 8*A*). Based on the composition of this gene locus, we speculated that these two genes form an operon.

We performed gene knockout of *Endo_dU* in *H. volcanii* (Fig. S12) by *pyrE*-based positive selection and increased the frequency of DNA deamination in cells by adding SB. The growth rate of the wild-type strain was slightly faster than the knockout strain without SB or 0.1 mM SB but grew as slowly as the knockout strain with 0.5 mM SB induction (Fig. S13). The addition of SB led to the growth arrest of both strains. The cell density in the stationary phase of knockout strains was lower than wild-type strains with or without SB induction. Total RNA was further extracted from cells for RNA sequencing, and TPM was used to estimate gene expression levels. The results showed that gene transcription levels of *Endo_dU*, *UDG,* and *EndoV* in wild-type strains were not obviously affected by SB (Fig. 8*B*). However, the *UDG* gene transcription level in *Endo_dU* knockout strain was significantly increased by 0.5 mM SB induction, however, the increased transcription of the *UDG* gene was not detected in low SB concentration (0.1 mM). These findings suggest that Endo_dU may play an important role in DNA repair induced by SB, together with UDG, because UDG will increase expression to compensate for the lack of Endo_dU. Taken together, Endo_dU may cooperate with UDG to process the misincorporation of uracil during DNA synthesis or the deaminated products of cytosine.

## Discussion

In this study, we investigated the evolution and enzymatic activity of DUF99 proteins which remained unknown for several decades. Based on the phylogenetic analysis and enzymatic characterization, we here showed that the DUF99 family is a novel endonuclease family of proteins that specifically recognize and cleave dU-containing DNA on the 3′-side of the deaminated base. We named this novel endonuclease family “Endonuclease dU” (Endo_dU).

The latest NCBI nr protein sequence database contains over 2000 Endo_dU homologs of archaeal and bacterial origin, with only two homologs from eukaryotes. Based on the phylogenetic analysis performed herein, Endo_dU proteins form a monophyletic group and do not cluster with other RNase H-like proteins, including EndoV. Among Endo_dU homologs, the bacterial proteins form a monophyletic group, closely related to the archaeal “TACK” superphylum homologs, distinct from other archaeal homologs. Notably, Endo_dU homologs from halophilic archaea belonging to the “Euryarchaeota” form a well-supported clade. Taken together, the Endo_dU-coding gene may have arisen in some prokaryotic lineages by horizontal gene transfer and selection, similar to other alternative endonucleases that are found in prokaryotes and involved in DNA repair (22, 23).

To determine the cleavage site of Endo_dU, we used 5′-FAM-labeled ssDNA substrate with dU at position 23 and tested its cleavage by five distinct Endo_dU proteins. The Endo_dU proteins specifically cleaved the dU-containing DNA strand, forming a 23-nt product. This indicated that the Endo_dU endonucleases cleave on the 3′-side of dU on DNA. This cleavage site has not been reported for other known endonucleases to date. Further, these enzymes specifically cleave the 3′-side of dU in a double-stranded substrate but do not cleave the complementary strand. Similarly, these enzymes do not cleave dsDNA substrates with a mismatched base pair. Taken together, the Endo_dU proteins have arisen as a novel endonuclease family that may be involved in DNA repair of uracil introduced into the genome by the misincorporation of uracil during DNA synthesis or by deamidation of cytosine.

Although most endonucleases require metal ions for function, AfuEndo_dU and HaloEndo_dU are active in the absence of metal ions, and even a high concentration of EDTA does not suppress their endonuclease activity. Similar metal-ion–independent nucleases, such as RNase L and RNase A (24, 25) have been previously identified. Furthermore, AfuEndo_dU and HaloEndo_dU are active over a wide temperature (4–55 °C) and pH range (4, 5, 6, 7, 8, 9, 10). Further, ionic strength impacts the endonuclease activity, with a maximum tolerated NaCl concentration of 200 mM. These moderate reaction conditions could be advantageous for *in vitro* applications of these enzymes, such as the introduction of a single nick on the 3′-side of dU on DNA. Notably, although AfuEndo_dU is derived from a thermophilic microorganism, recombinant AfuEndo_dU precipitated after total cell lysate incubation at 65 °C for 20 min. Further, the recombinant AfuEndo_dU protein purified in this study showed no endonuclease activity at high temperatures. These observations suggest that recombinant AfuEndo_dU expressed in *E. coli* lack extrinsic factors required for its thermostability (26), indicating that AfuEndo_dU is probably not an intrinsically thermostable protein.

Previous studies showed SB is a base deamination inducer in bacteria (27, 28). Analysis of the Endo_dU-encoding genomes revealed an apparent colocalization of *Endo_dU* and *UDG* genes in halophiles, probably under the control of the same promoter. We further deleted a part of the middle region of *Endo_dU* gene in *H. volcanii* genome and found the growth rate and the cell density in the stationary phase of the knockout strain was lower. The results of RNA sequencing revealed that transcription levels of Endo_dU, UDG, and EndoV were not affected by 0.5 mM SB in wild-type strain. However, the transcription level of the *UDG* gene in *Endo_dU* knockout strain largely increased when induced by 0.5 mM SB. Since we only deleted a part of the middle region of *Endo_dU* gene and retained the upstream promoter, we speculate that Endo_dU and UDG function coordinately in wild-type strain more efficiently than a single gene alone. The lack of Endo_dU makes cells express more UDG to overcome the base deamination, and the expression levels of diverse DNA repair nucleases together are sufficient to overcome the base deamination in wild-type strains naturally. Multiple alternative endonucleases involved in uracil repair have also been identified in other studies, *e.g.*, ExoIII homolog Mth212 from *Methanothermobacter thermautotrophicus* (29). Accordingly, we speculate that Endo_dU mediates another novel DNA repair pathway that replaces or complements the conventional uracil repair pathways, especially in extremophiles that have to survive under stressful conditions. However, the processes driving the selection of different repair mechanisms during the evolution of individual species are currently unclear.

We also analyzed the sequence and structural features of Endo_dU family to better understand the cleavage mechanism. The structure of AfuEndo_dU contains a typical RNase H-like fold, characteristic of one of the clans of 64 RNase H-like protein families. Using structure comparison and conservation analysis, we identified a putative conserved uracil-recognition pocket, surrounded by motifs IV to VII. In addition, the sequence and structure analyses revealed little similarity shared by some regions of Endo_dU and EndoV, with the conserved DEDD motif found in most RNase H-like enzymes incomplete in Endo_dU. Only half of the analyzed Endo_dU enzymes possess the first aspartic acid residue in βA, which is considered to be conserved in most RNase H-like enzymes (21). Notably, in Endo_dU, a conserved aspartic acid or glutamic acid residue is adjacent (to the right) to this aspartic acid residue; however, its substitution did not affect the endonuclease activity. A similar phenomenon was reported for Prp8, which also possesses an incomplete DEDD active site (21, 30). Further, the substitution of D10 and H166 abolished the endonuclease activity of AfuEndo_dU, suggesting that these two residues are critical for enzyme function. Taken together, these preliminary analyses revealed that Endo_dU may utilize a novel catalytic mechanism to recognize and cleave dU-containing DNA. However, further experiments, focused on solving the structure of Endo_dU complexes with a DNA substrate to understand the cleavage mechanism, are still needed in the future.

In conclusion, the above findings establish a novel endonuclease family and investigate its enzymatic properties. However, the details of the cleavage mechanism and downstream processes from the Endo_dU pathway remain to be determined. Moreover, Endo_dU proteins could be developed into new tools for *in vitro* applications, such as the one-step introduction of a single nick 3′ to dU on DNA.

## Experimental procedures

### Synthetic DNA substrates

dI, dU and AP site-containing oligonucleotides, 5′-carboxyfluorescein (FAM) fluorescently labeled oligonucleotides, and gene-specific primers used for gene knockout were generated by chemical synthesis at Beijing Genomics Institute or Tsingke Biological Technology. Further, dsDNA substrates were generated by mixing the complementary strands at 1:1 M ratio in an annealing buffer [20 mM Tris-HCl, pH 7.4, and 150 mM NaCl], heated at 95 °C for 3 min, and cooling down 0.1 °C every 8 s until 25 °C. The sequences of all oligonucleotides used in this study are provided in Table S1.

### Phylogenetic analysis of DUF99 proteins

The amino acid sequence encoding DUF99 protein of *A. fulgidus* (NCBI accession no. WP_010878930.1) was used as a query in Basic Local Alignment Search Tool (BLAST) search (31) of a non-redundant (nr) protein sequence database at the National Center for Biotechnology Information (NCBI; http://www.ncbi.nlm.nih.gov). Similar sequences (E-value < 0.05) were downloaded from GenBank (ftp://ftp.ncbi.nih.gov/genomes/genbank) in May 2021. Representative amino acid sequences of DUF99 and several RNAase H-like domain-containing proteins, such as EndoV, UvrC, and RNase HII were aligned to profile from previous study (21) using MAFFT (version 7.310, settings: --keeplength) (32). Maximum-likelihood tree was then inferred using IQ-TREE (version 2.0.7) (33) based on LG + G model with 1000 ultrafast bootstrap (34, 35). For phylogenetic analysis of DUF99 proteins alone, the non-redundant DUF99 protein sequences were aligned using MUSCLE program (version 3.8.31) (36) and trimmed using trimAL (version 1.2) (37). The maximum-likelihood phylogenetic tree was then constructed using IQ-TREE with 1000 ultrafast bootstrap alignments. The iTOL software (version 6) (38) was used for tree visualization.

### Protein expression and purification

DUF99 proteins from *A. fulgidus* (WP_010878930.1), *Halobacterium* sp. DL1 (NCBI accession no. WP_009487363.1), Gammaproteobacteria bacterium (NCBI accession no. TLY50285.1), *Candidatus* Lokiarchaeota archaeon (NCBI accession no. TFG01626.1), and *Candidatus* Thorarchaeota archaeon (NCBI accession no. TFG14500.1) were chemically synthesized as His-tag–containing constructs (Tsingke) and cloned into pCold II plasmid (Takara) after codon-optimization for expression in *E. coli*. Mutated encoding sequences were also obtained by direct chemical synthesis. All plasmids were further validated by sanger sequencing using sequencing primers (Table S1) by the Beijing Genomics Institution.

For recombinant protein production, *E. coli* BL21 cells harboring the corresponding plasmids were cultured in Luria–Bertani (LB) medium containing 100 μg/ml ampicillin at 37 °C until OD_600_ of 0.8. Recombinant protein expression was induced by the addition of isopropyl β-d-1-thiogalactopyranoside to a final concentration of 0.2 mM and incubating overnight at 15 °C. The cells were then collected by centrifugation and resuspended in wash buffer [50 mM Tris-HCl, pH 7.4, 300 mM NaCl, 0.5 mM tris(2-carboxyethyl)phosphine (TCEP), and 20 mM imidazole] supplemented with 1 mM phenylmethylsulfonyl fluoride (PMSF) and protease inhibitor cocktail (Sangon). The cells were lysed by sonication. For proteins that might be thermophilic, the lysate was incubated at 65 °C for 20 min before centrifugation. Lysate containing soluble proteins was obtained by centrifugation at 12,000 rpm for 10 min and was applied onto a HisTrap HP column (GE Healthcare). The column was washed extensively with the wash buffer, and the bound protein was eluted with the wash buffer containing 300 mM imidazole. The eluted peak fractions were pooled and dialyzed into storage buffer [50 mM Tris-HCl, pH 7.4, 300 mM NaCl, 0.5 mM TCEP, and 10% (vol/vol) glycerol] by ultrafiltration using an Amicon Ultra 3K device (Millipore), flash-frozen in liquid nitrogen, and stored at −80 °C. The protein concentrations were determined with the BCA Protein Assay Kit (Beyotime), using bovine serum albumin (BSA) protein to create the standard curve, and plotting the average 562 nm measurement for each BSA standard and our protein samples.

### Determination of cleavage substrates and cleavage site

300 nM proteins were individually incubated with 30 nM nucleic acids substrate in reaction buffer A [20 mM Tris-HCl, pH 7.4, 150 mM NaCl, 5 mM MgCl_2_, 2 mM dithiothreitol (DTT), and 5% (vol/vol) glycerol] at 37 °C for 30 min. Reactions were quenched by the addition of an equal volume of formamide gel loading buffer [95% formamide, 5 mM ethylenediaminetetraacetic acid (EDTA), 0.5% sodium dodecyl sulfate and 0.025% bromophenol blue]. Cleavage products were heated at 95 °C for 5 min, chilled on ice, and resolved on 8 M urea–15% denaturing polyacrylamide gel electrophoresis (PAGE) in TBE buffer [89 mM Tris, 89 mM boric acid and 2 mM EDTA, pH 8.3]. Bands were visualized using the ChemiDoc gel imaging system (Bio-Rad). All cleavage reactions were repeated at least in three independent experiments.

### Optimization of cleavage conditions

To investigate the requirement for divalent metal cations, cleavage experiments were performed using 300 nM proteins and 30 nM DNA substrate in reaction buffer B [20 mM Tris-HCl, pH 7.4, 150 mM NaCl, 1 mM DTT, and 5% (vol/vol) glycerol] supplemented with 5 mM MgCl_2_, MnCl_2_, CoCl_2_, CuCl_2_, ZnCl_2_, CaCl_2_, NiCl_2_, or EDTA. To explore the effect of ionic strength, cleavage reactions were performed using 300 nM proteins and 30 nM DNA substrate in reaction buffer C [20 mM Tris-HCl, pH 7.4, 1 mM DTT, and 5% (vol/vol) glycerol] supplemented with 50, 100, 200, 400, 800 or 1000 mM NaCl. The effect of pH was tested using 300 nM proteins and 30 nM DNA substrate in reaction buffer B with a pH range of 5 to 10 pH units. The cleavage reactions above were incubated at 37 °C for 30 min.

To determine the optimal reaction temperature, reaction mixtures with 300 nM proteins and 30 nM DNA substrate in reaction buffer B were incubated at the assayed temperature for 30 min. Endo_dU thermostability was evaluated by heating the proteins at 37 °C or 65 °C for different periods of time before assaying at 37 °C. All cleavage products were resolved on 8 M urea–15% denaturing PGAE and visualized using ChemiDoc gel imaging system (Bio-Rad).

### Sequences and structural analysis of Endo_dU

The crystal structures of AfuEndo_dU and *Thermotoga maritima* EndoV (TmEndoV) (39) were downloaded from the Protein Data Bank (http://www.rcsb.org/pdb) (accession numbers 2QH9 and 2W35, respectively). The structure of HaloEndo_dU was predicted by using AlphaFold2 (40). The ConSurf server (41) was used to calculate Endo_dU sequence conservation. Structure superposition and all structural figures were created using PyMOL (version 2.2.0; Schrödinger, LLC).

### *H. volcanii* cell cultivation, gene knockout, and RNA sequencing

*H. volcanii* H26 (ΔpyrE2) cells were grown at 45 °C in Hv-YPC medium containing, per liter, 1 g casamino acids, 1 g peptone, 5 g yeast extract, 21 g MgSO_4_·7H_2_O, 18 g MgCl_2_·6H_2_O, 4.2 g KCl, 144 g NaCl and 12 mM Tris-HCl (pH 7.5). Gene knockout of a part of the middle region of *Endo_dU* gene was carried out by homologous recombination as previously described (42, 43). Briefly, flanking regions of 500 nt upstream and downstream of *Endo_dU* gene, and 500 nt of 3′ region of *Endo_dU* gene were amplified by PCR using gene-specific primers (Table S1). The PCR products were fused with *pyrE* gene derived from pTA131 plasmid *via* gibson assembly, forming a fragment of “500 nt 3′ region–pyrE–500 nt upstream–500 nt downstream”. The gene fragment was then amplified by PCR and transformed 1 μg into *H. volcanii* H26 by using polyethylene glycol 600 as described previously (44). Finally, two successive rounds of selection on Hv-Ca plates containing, per liter, 5 g casamino acids, 21 g MgSO_4_·7H_2_O, 18 g MgCl_2_·6H_2_O, 4.2 g KCl, 144 g NaCl, 15g agar and 12 mM Tris-HCl (pH 7.5), and Hv-Ca plates containing 50 mg/ml 5-fluoroorotic acid were used to select the positive clones with *Endo_dU* gene deletion. Genomic DNA of *H. volcanii* strains was extracted using DNeasy PowerSoil Pro Kit (Qiagen) according to the manufacturer’s instructions. Gene knockout of *Endo_dU gen*e on *H. volcanii* was further validated by Sanger sequencing using gene-specific primers (Table S1 and Fig. S11).

To induce DNA deamination, wild type and knockout strains were grown in Hv-YPC medium at 45 °C and continuously monitored the OD_600_. When OD_600_ reached about 0.5, SB was added to the final concentration of 0.1 or 0.5 mM and continuously cultured for 48 h. Cells were harvested by centrifugation at 12,000 rpm at 4 °C for 20 min. After that, total RNA was extracted immediately using Direct-zolRNA MiniPrep Kits (Zymo), according to the manufacturer’s instructions. Ribosomal RNA was removed using Ribo-Zero rRNA Removal Kit (Epicentre). Sequencing libraries were generated using NEBNext Ultra II Directional RNA Library Prep Kit for Illumina (NEB) following the manufacturer’s recommendations, and sequenced by Magigene Biotechnology using Illumina NovaSeq6000 PE150 platform.

Raw reads were quality filtered and adapter trimmed with fastp (45). Trimmed reads were then mapped to the genome of *H. volcanii* DS2 using Bowtie2 (46). After mapping, normalized expression metrics such as Reads Per Kilobase Million (RPKM), Fragments Per Kilobase Million (FPKM), and Transcripts Per Million (TPM) were calculated using StringTie (47).

## Data availability

Sequencing reads were deposited at the National Center for Biotechnology Information BioProject with accession number PRJNA1072706. Other data associated with this manuscript can be found in the supporting information file.

## Supporting information

This article contains supporting information. Supplementary Figure S1-S13 and Table S1.

## Conflict of interest

The authors declare that they have no conflicts of interest with the contents of this article.
