# Supplementary material for: DUF99 family proteins are novel endonucleases that cleave deoxyuridine on DNA substrates
Source: J Biol Chem. 2024 Oct 18;300(11):107901. doi: 10.1016/j.jbc.2024.107901 (PMC11585767; doi:10.1016/j.jbc.2024.107901)
Supplement: Supporting information [file mmc1.pdf]

Supporting information

DUF99 family proteins are novel endonucleases that cleave deoxyuridine on DNA substrates

Jinquan Li[1], Runyue Xia[1], Wen-Cong Huang[1,2], Jiazheng Gu[1] and Meng Li[1*]

[1] Archaeal Biology Centre, Synthetic Biology Research Center, Shenzhen Key Laboratory of Marine Microbiome Engineering, Key Laboratory of Marine Microbiome Engineering of Guangdong Higher Education Institutes, Institute for Advanced Study, Shenzhen University, Shenzhen 518060, China

[*] For correspondence: Meng Li, limeng848@szu.edu.cn

[2] Present address: Department of Marine Microbiology and Biogeochemistry, NIOZ, Royal Netherlands Institute for Sea Research, 1790 AB Den Burg, Netherlands

Supplementary Figures

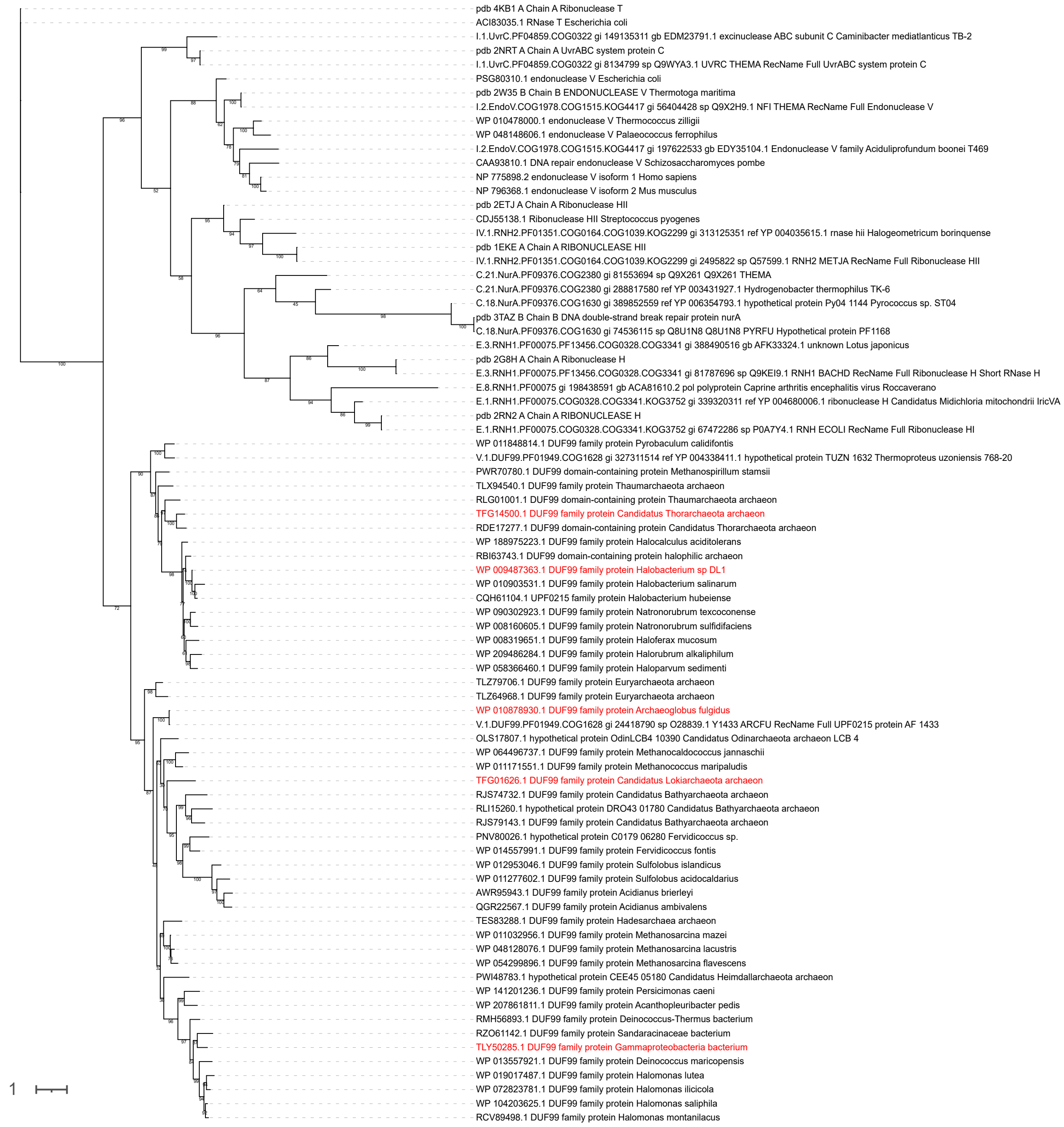

Supplementary Figure S1. Maximum-likelihood phylogenetic tree of DUF99 proteins with representative RNase H-like domain containing proteins related to Figure 1A. The phylogenetic tree was inferred using IQ-TREE with LG+G model and aligned sequences as indicated. RNase T was taken as an outgroup. Bootstrap values are shown at each node. DUF99 proteins chosen for enzymatic activity analysis are shown in red.

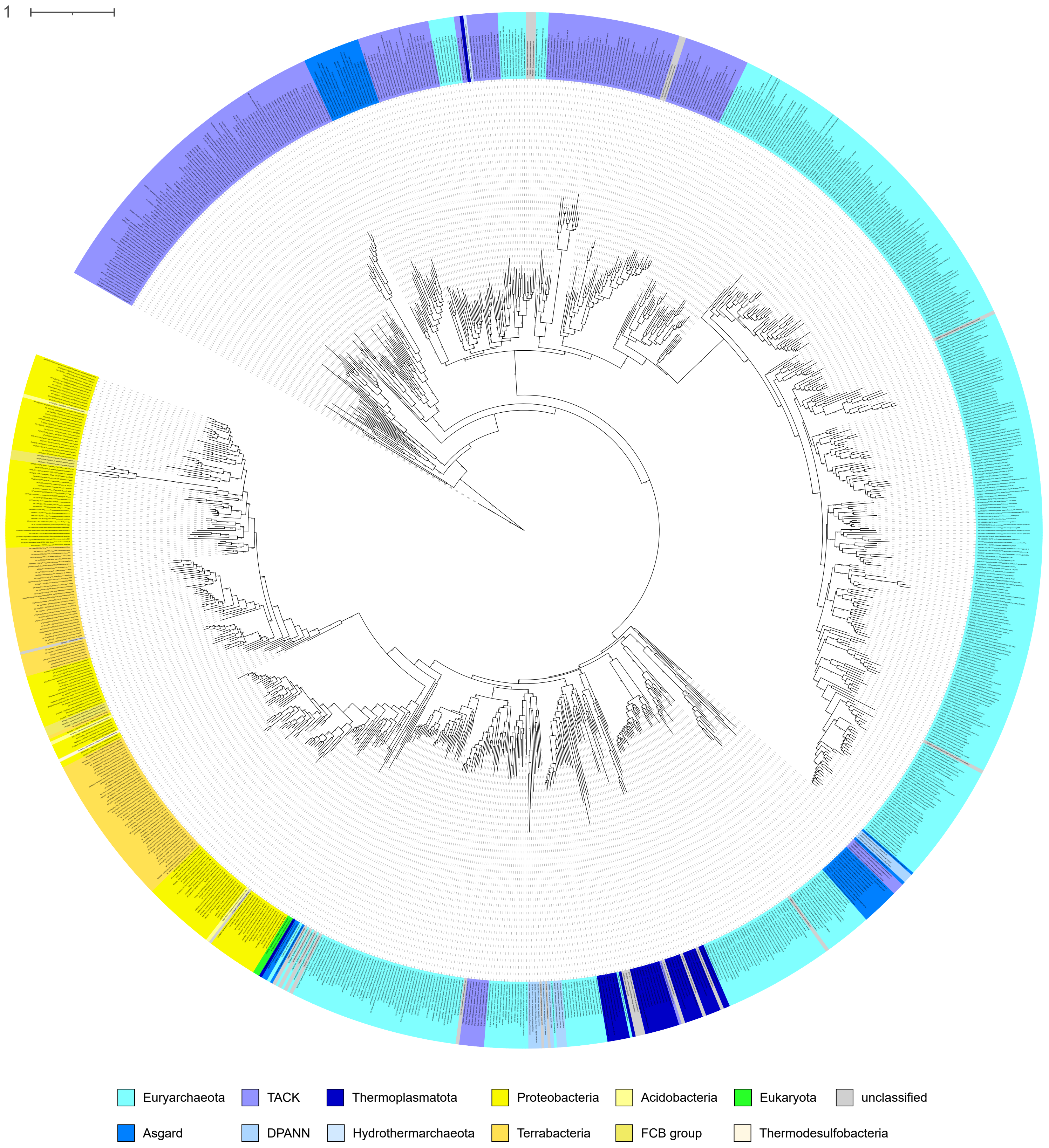

Supplementary Figure S2. Circular maximum-likelihood phylogenetic tree of DUF99 proteins. The phylogenetic tree was generated using sequences from Figure 1B. Bootstrap values larger than 70 are shown. Labels are colored according to taxonomy shown in the legend.

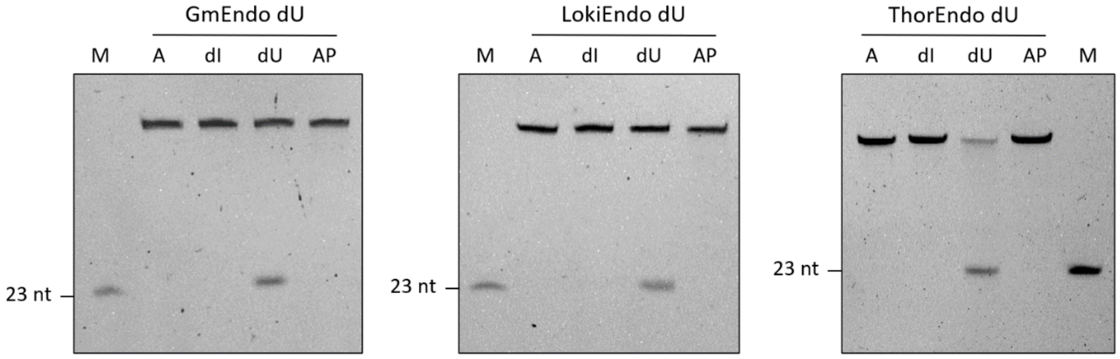

Supplementary Figure S3. Cleavage activities of three other Endo_dU homologs toward different ssDNA substrates. Endo_dU of Gammaproteobacteria bacterium (GmEndo_dU), *Candidatus* Lokiarchaeota archaeon (LokiEndo_dU) and *Candidatus* Thorarchaeota archaeon (ThorEndo_dU) were incubated with 30 nM 5′-FAM-labeled ssDNA substrates at 37°C for 30 min individually.

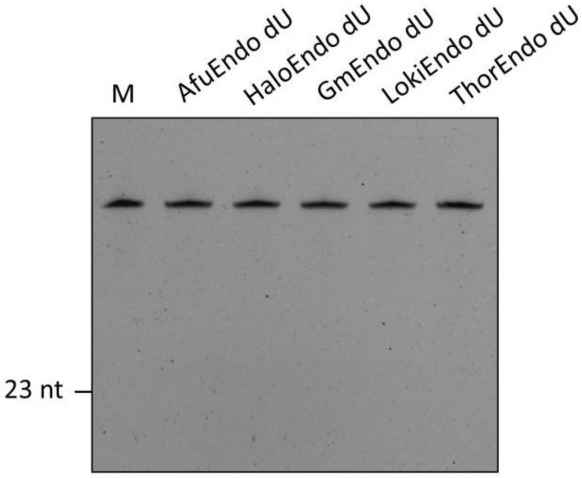

Supplementary Figure S4. Cleavage of ssRNA substrate by Endo_dU. Five Endo_dU homologs were incubated with 30 nM 5′-FAM-labeled ssRNA substrates at 37°C for 60 min. The cleavage products were resolved by 8 M urea–15% denaturing PGAE.

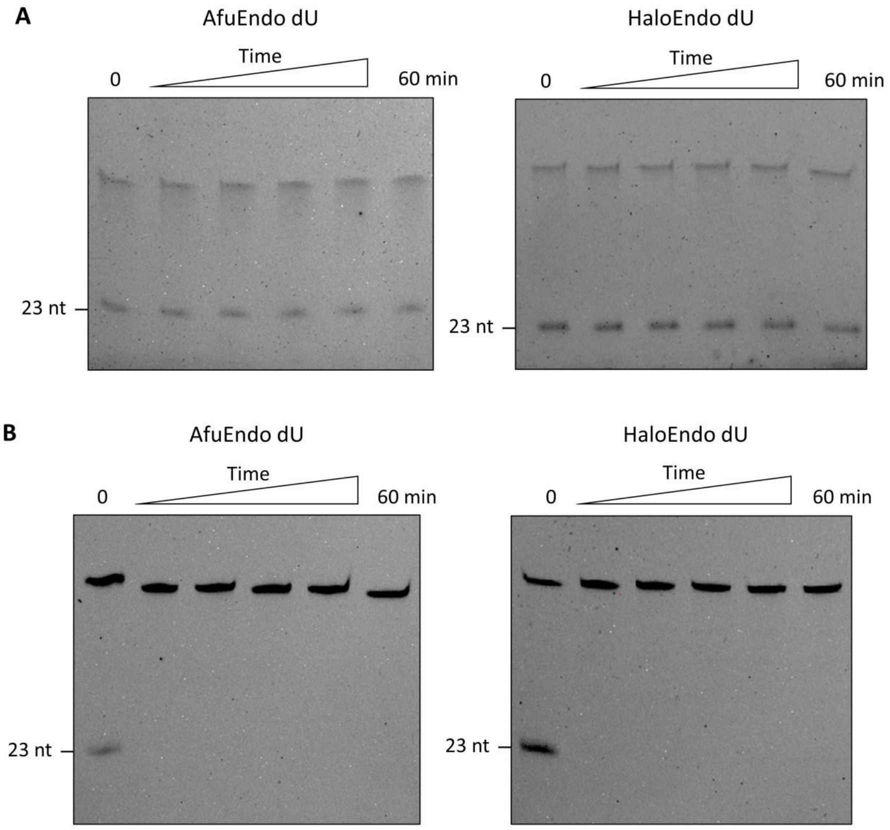

Supplementary Figure S5. Thermostability of AfuEndo_dU and HaloEndo_dU. (**A**) Proteins were incubated at 37°C for increasing time (0, 10, 20, 30, 40, 50 min) prior to cleavage reactions. (**B**) Proteins were incubated at 65°C for increasing time (0, 10, 20, 30, 40, 50 min) prior to cleavage reactions. The final reaction mixtures were performed at 37°C for 60 min.

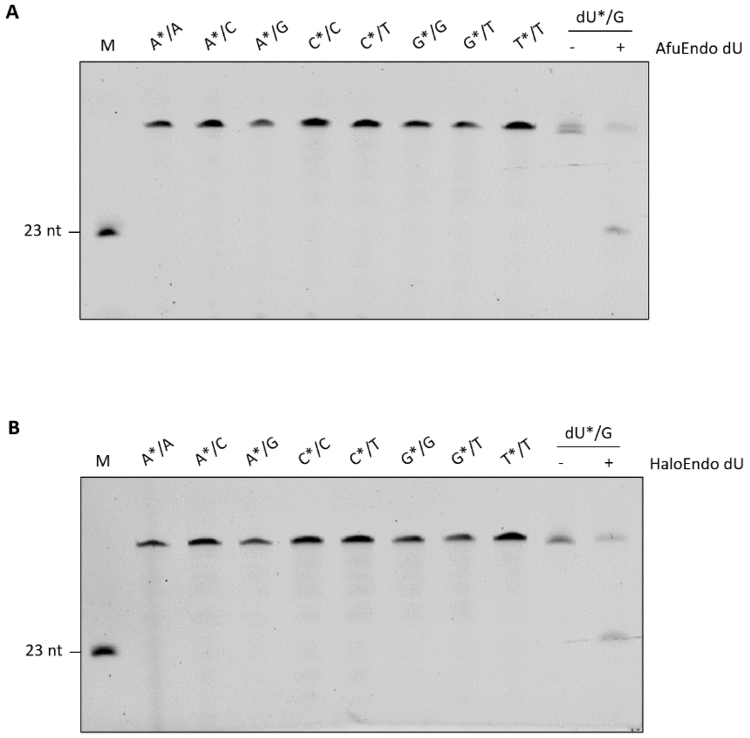

Supplementary Figure S6. Endonuclease activities of Endo_dU toward dsDNA substrates containing a mismatched base pair. (**A**) AfuEndo_dU and (**B**) HaloEndo_dU (300 nM) cleaved the indicated dsDNA substrates (30 nM) at 37°C for 60 min. The mismatched base pair at position 23 are showed above, and the labeled strands are indicated by asterisks. M, 23 nt ssDNA marker

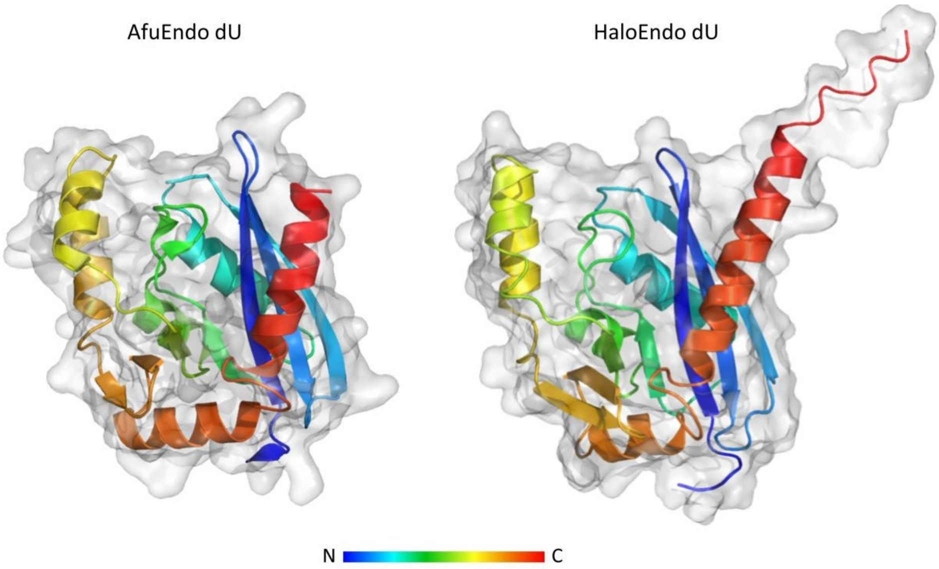

Supplementary Figure S7. Structures of AfuEndo_dU and HaloEndo_dU. The structures are shown in the ribbon representation (rainbow color, blue at the N-terminus to red at the C-terminus) and superposition of surface representation (gray color).

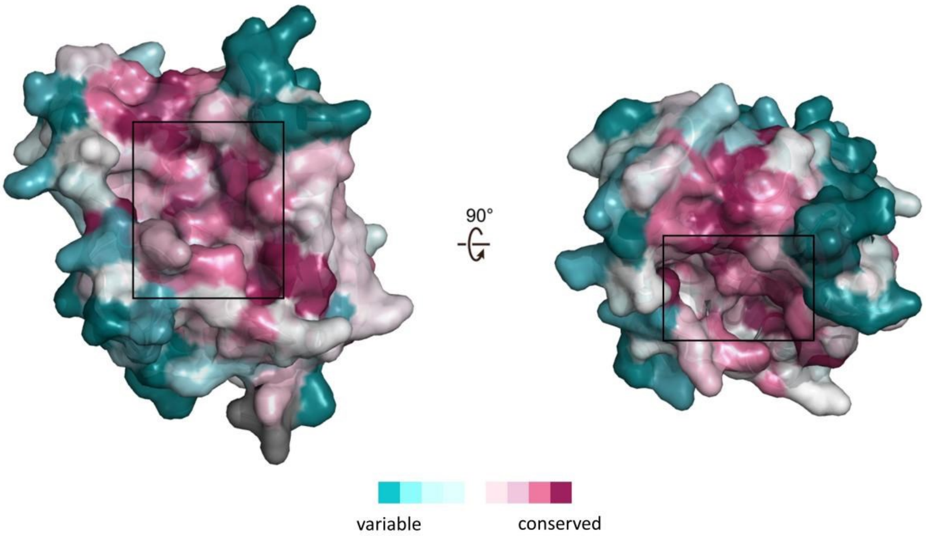

Supplementary Figure S8. Putative uracil-binding pocket of Endo_dU. Conservation of the putative uracil-binding pocket is shown in surface representation, colored from cyan (highly variable regions) to magenta (highly conserved regions). The putative pocket is highlighted by the black box.

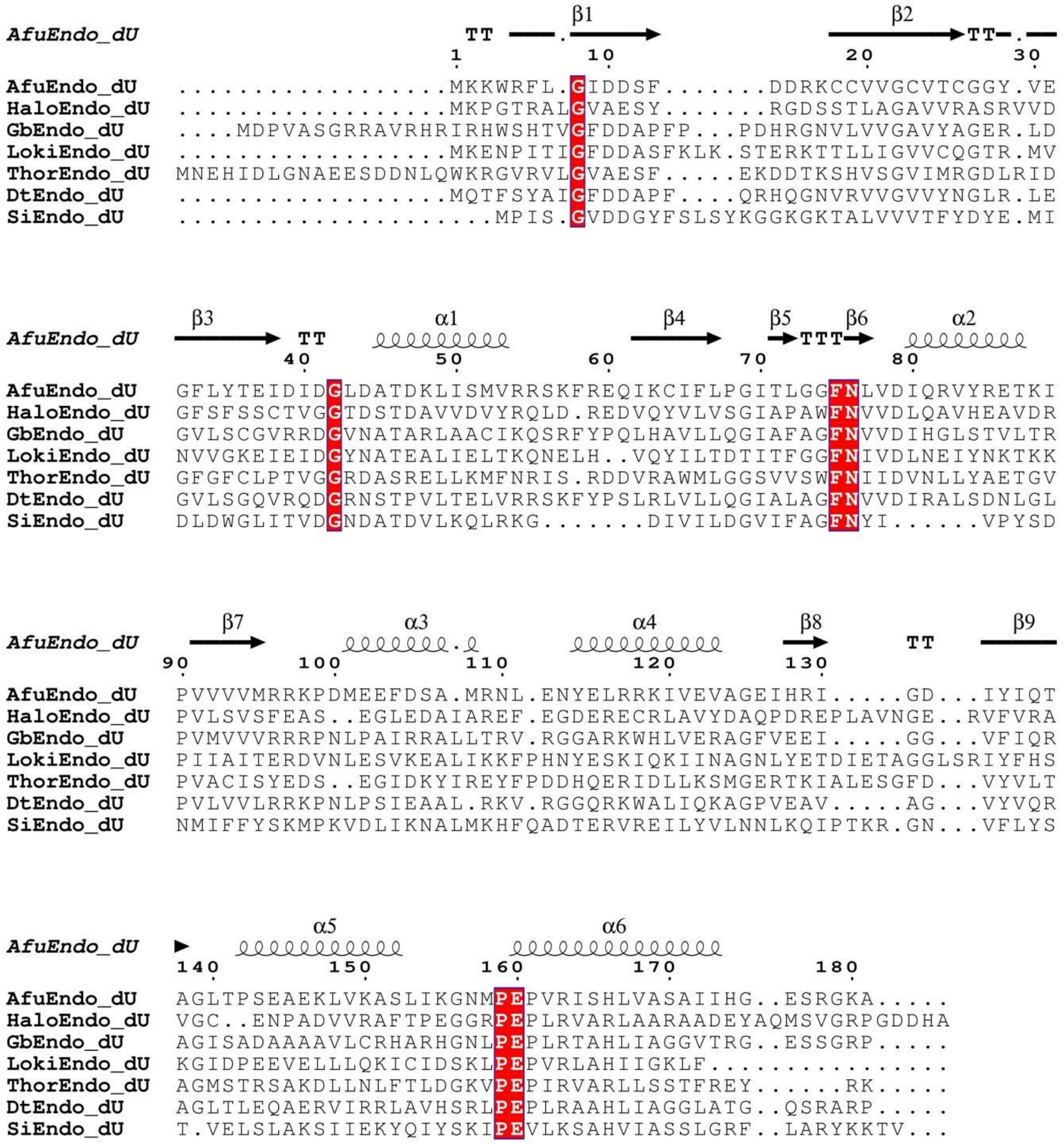

Supplementary Figure S9. Multiple sequence alignment of the selected Endo_dU related to Figure 7A. The secondary structure elements of Endo_dU is indicated above the sequences, using the structure of AfuEnod_dU as the template. The figure was visualized using ESPript3 (http://espript.ibcp.fr/ESPript/ESPript).

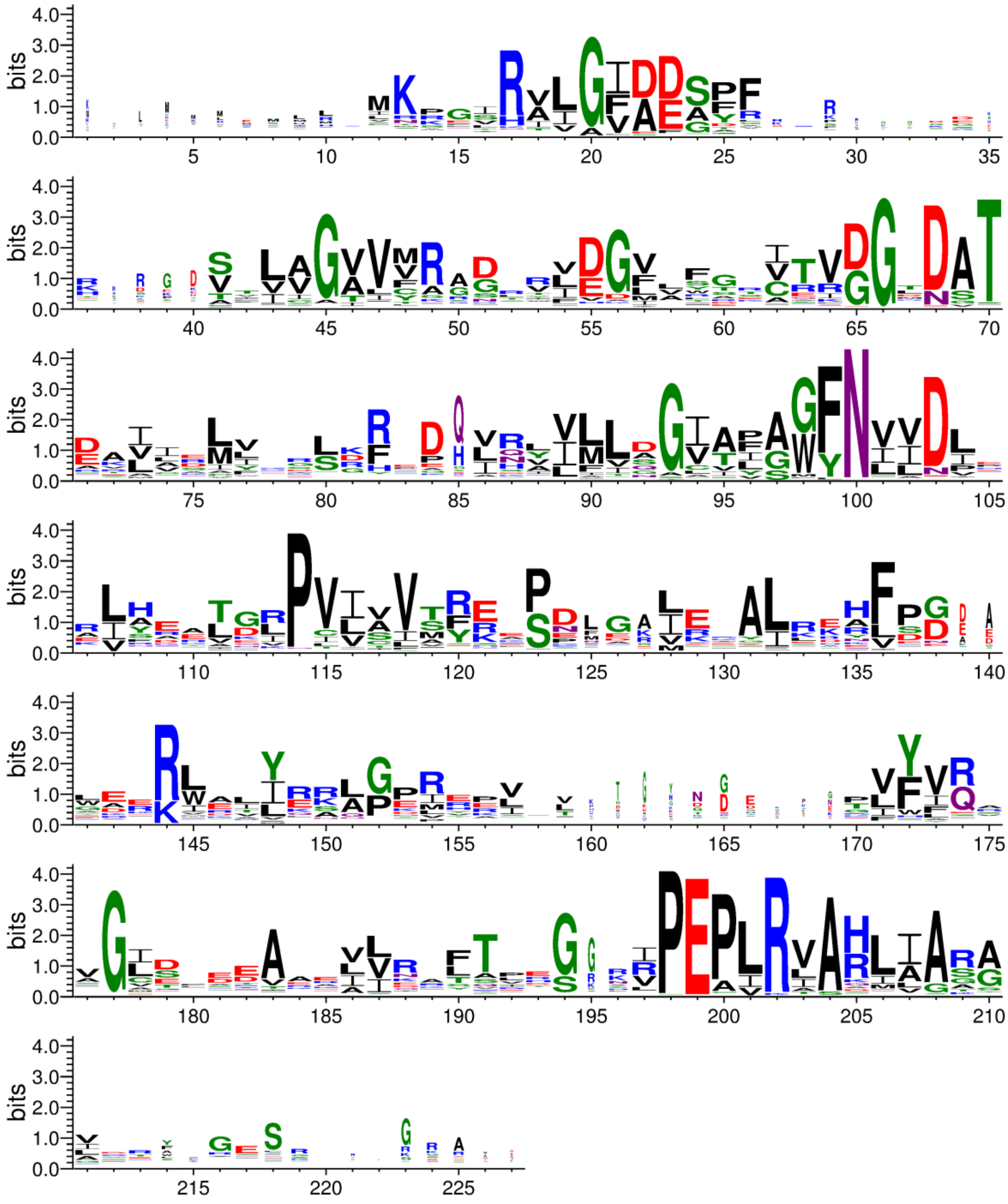

Supplementary Figure S10. WebLogo analysis of full-length Endo_dU related to Figure 7D. Sequence logos were generated using the multiple sequence alignment result from the phylogenetic analysis of DUF99 proteins, and visualized by WebLogo tool (http://weblogo.threeplusone.com).

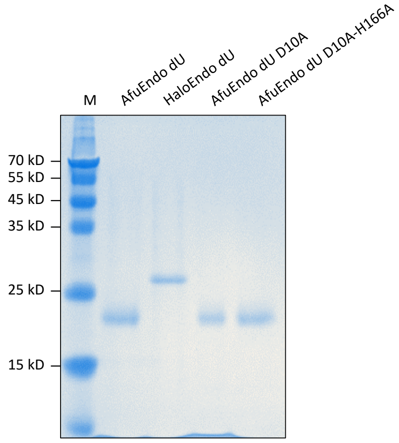

Supplementary Figure S11. 12.5% SDS-PAGE analysis of AfuEndo_dU, HaloEndo_dU, and AfuEndo_dU mutant proteins. M, molecular weight marker. The gel was stained with Coomassie Brilliant Blue.

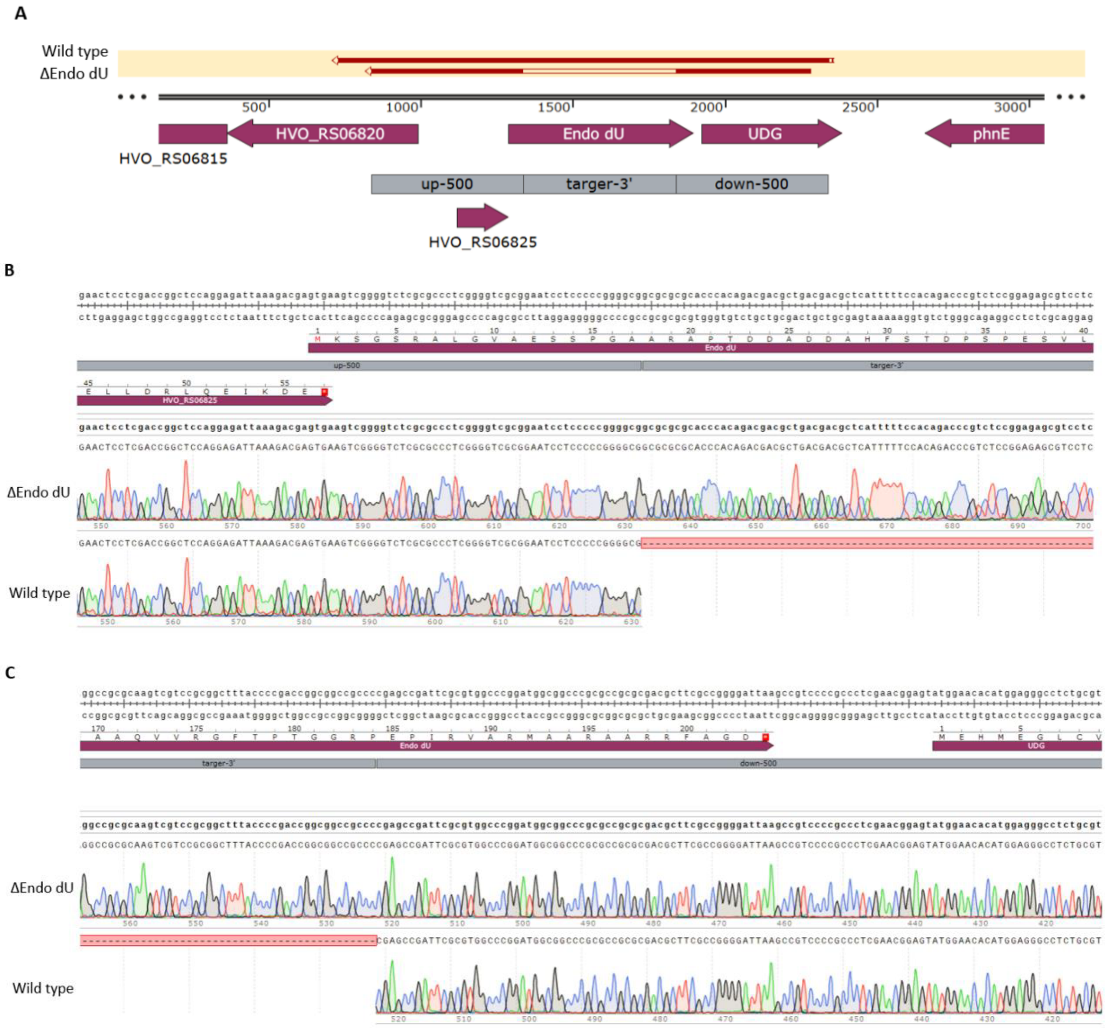

Supplementary Figure S12. Sanger DNA sequencing of wild type and *ΔEndo_dU H. volcanii* strains. (A) Overview of DNA sequence alignment of sequence reads and DNA genome nearby the *Endo_dU* region. Red regions on the top are fully aligned, while hollow regions are mismatched. (B) Zoom in the alignment of *Endo_dU* upstream region. (C) Zoom in the alignment of *Endo_dU* downstream region.

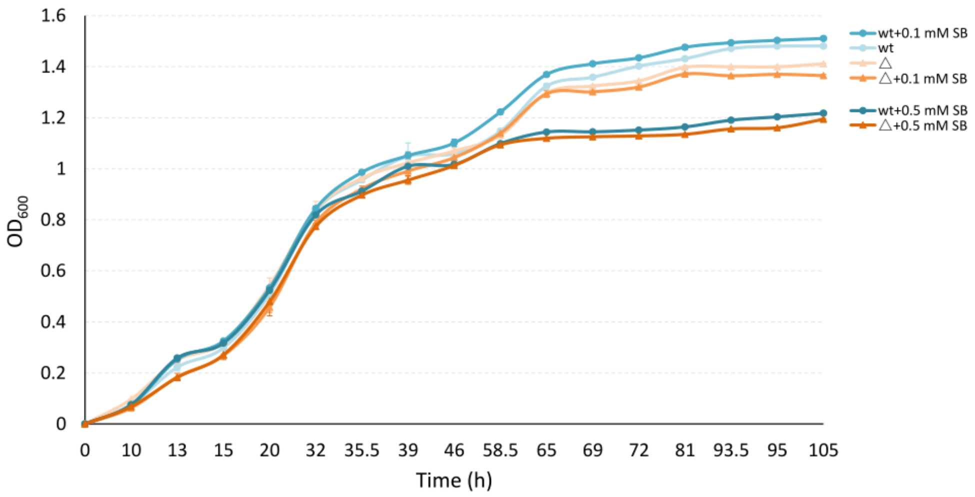

Supplementary Figure S13. Growth curves of wild type (wt) and *ΔEndo_dU* (Δ) *H. volcanii* strains with different concentrations of SB induction. SB was added after 20 h culture when OD_{600} reach about 0.5. Data points represent mean ± SEM of three biological replicates (n=3).

Supplementary Table

Supplementary Table S1. Sequences of oligonucleotides used in this study.

Name	Sequence (5′-3′)	Description
ssDNA-23dU	CACCAGTAGCACCATTACCATT(dU)GCAAGGCC GGAAACGTCACCAATGAAACCATCGAT	dU-containing ssDNA substrate, dU at position 23
ssDNA-23dI	CACCAGTAGCACCATTACCATT(dI)GCAAGGCCG GAAACGTCACCAATGAAACCATCGAT	dI-containing ssDNA substrate, dI at position 23
ssDNA-23AP	CACCAGTAGCACCATTACCATT(AP)GCAAGGCC GGAAACGTCACCAATGAAACCATCGAT	AP-containing ssDNA substrate, AP site at position 23
ssDNA-23A	CACCAGTAGCACCATTACCATTAGCAAGGCCGG AAACGTCACCAATGAAACCATCGAT	undamaged ssDNA substrate, A at position 23
ssDNA-23T	CACCAGTAGCACCATTACCATTTGCAAGGCCGG AAACGTCACCAATGAAACCATCGAT	undamaged ssDNA substrate, T at position 23
ssDNA-23C	CACCAGTAGCACCATTACCATTCGCAAGGCCGG AAACGTCACCAATGAAACCATCGAT	undamaged ssDNA substrate, C at position 23
ssDNA-23G	CACCAGTAGCACCATTACCATTGGCAAGGCCGG AAACGTCACCAATGAAACCATCGAT	undamaged ssDNA substrate, G at position 23
ssRNA	CACCAGUAGCACCAUUACCAUUAGCAAGGCCG GAAACGUCACCAAUGAAACCAUCGAU	ssRNA substrate
50nt-23dU	CACCAGTAGCACCATTACCATT(dU)GCAAGGCC GGAAACGTCACCAATGAAA	50-nt dU-containing ssDNA substrate, dU at position 23
35nt-23dU	CACCAGTAGCACCATTACCATT(dU)GCAAGGCC GGAA	35-nt dU-containing ssDNA substrate, dU at position 23
22nt-marker	CACCAGTAGCACCATTACCATT	22 nt ssDNA marker
23nt-marker	CACCAGTAGCACCATTACCATT(dU)	23 nt ssDNA marker
24nt-marker	CACCAGTAGCACCATTACCATT(dU)G	24 nt ssDNA marker
BTM-A	ATCGATGGTTTCATTGGTGACGTTTCCGGCCTTG CAAATGGTAATGGTGCTACTGGTG	complementary strand to ssDNA-23T
BTM-T	ATCGATGGTTTCATTGGTGACGTTTCCGGCCTTG CTAATGGTAATGGTGCTACTGGTG	complementary strand to ssDNA-2A
BTM-C	ATCGATGGTTTCATTGGTGACGTTTCCGGCCTTG CCAATGGTAATGGTGCTACTGGTG	complementary strand to ssDNA-23G
BTM-G	ATCGATGGTTTCATTGGTGACGTTTCCGGCCTTG CGAATGGTAATGGTGCTACTGGTG	complementary strand to ssDNA-23C
pCold_F	ACGCCATATCGCCGAAAGG	forward primer to verify the expression plasmids
pCold_R	GGCAGGGATCTTAGATTCTG	reverse primer to verify the expression plasmids
Hv-up500_F	CTATTGGTTAAAAAATGAGCTGGACCACGGCGT	forward primer to amplify

	CGAGGTC	500 nt upstream of Endo_dU gene
Hv-up500_R	CCGGGCCACGCGAATCGGCTCGCGCCCCGGGGGAGG	reverse primer to amplify 500 nt upstream of Endo_dU gene
Hv-down500_F	CGCGGAATCCTCCCCCGGGGCGCGAGCCGATTCGCGTGG	forward primer to amplify 500 nt downstream of Endo_dU gene
Hv-down500_R	CGGACTCGTTCGTGATGGC	reverse primer to amplify 500 nt downstream of Endo_dU gene
Hv-target3'_F	GCGCGCGCACCCAC	forward primer to amplify 3' region of Endo_dU gene
Hv-target3'_R	ATTAAAGAACGTGGACTCCAACGGGCGGCCGCCG	reverse primer to amplify 3' region of Endo_dU gene
pTA131-pyrE_F	TTACCCCGACCGGCGGCCGCCCGTTGGAGTCCACGTTCTTTAATAGTGG	forward primer to amplify pyrE gene from pTA131
pTA131-pyrE_R	CGACGACCTCGACGCCGTGGTCCAGCTCATTTTTTAACCAATAGGCCG	reverse primer to amplify pyrE gene from pTA131
Hv_F	TGTAGACCACTTCGAACGACTC	forward primer to verify the positive clones
Hv_R	GAGGAGGACGCGGTAAGAG	reverse primer to verify the positive clones
